# Machine learning radiomics of magnetic resonance imaging predicts recurrence-free survival after surgery and correlation of LncRNAs in patients with breast cancer: a multicenter cohort study

**DOI:** 10.1186/s13058-023-01688-3

**Published:** 2023-11-01

**Authors:** Yunfang Yu, Wei Ren, Zifan He, Yongjian Chen, Yujie Tan, Luhui Mao, Wenhao Ouyang, Nian Lu, Jie Ouyang, Kai Chen, Chenchen Li, Rong Zhang, Zhuo Wu, Fengxi Su, Zehua Wang, Qiugen Hu, Chuanmiao Xie, Herui Yao

**Affiliations:** 1 Guangdong Provincial Key Laboratory of Malignant Tumor Epigenetics and Gene Regulation, Department of Medical Oncology, Breast Tumor Center, Phase I Clinical Trial Centre, Artificial Intelligence Laboratory, Sun Yat-Sen Memorial Hospital, Sun Yat-Sen University, No. 107 Yanjiang West Road, 510120 Guangzhou, People’s Republic of China; 2 Faculty of Medicine, Macau University of Science and Technology, Taipa, Macao People’s Republic of China; 3 Department of Medical Oncology, The Third Affiliated Hospital of Sun Yat-Sen University, Guangzhou, People’s Republic of China; 4 Imaging Diagnostic and Interventional Center, Sun Yat-Sen University Cancer Center, State Key Laboratory of Oncology in South China, Collaborative Innovation Center for Cancer Medicine, No. 651 Dongfeng East Road, Guangzhou, Guangdong People’s Republic of China; 5 Department of Breast Surgery, Dongguan Tungwah Hospital, Dongguan, People’s Republic of China; 6 Department of Radiology, Shunde Hospital, Southern Medical University, No. 1 Jiazi Road, Lunjiao Town, Shunde District, Foshan, 528300 People’s Republic of China; 7 Division of Science and Technology, Beijing Normal University-Hong Kong Baptist University United International College, Hong Kong Baptist University, Zhuhai, People’s Republic of China

**Keywords:** Machine learning, Radiomics, Magnetic resonance imaging, Recurrence-free survival, Treatment decisions, Long non-coding RNAs, Breast cancer

## Abstract

**Background:**

Several studies have indicated that magnetic resonance imaging radiomics can predict survival in patients with breast cancer, but the potential biological underpinning remains indistinct. Herein, we aim to develop an interpretable deep-learning-based network for classifying recurrence risk and revealing the potential biological mechanisms.

**Methods:**

In this multicenter study, 1113 nonmetastatic invasive breast cancer patients were included, and were divided into the training cohort (*n* = 698), the validation cohort (*n* = 171), and the testing cohort (*n* = 244). The Radiomic DeepSurv Net (RDeepNet) model was constructed using the Cox proportional hazards deep neural network DeepSurv for predicting individual recurrence risk. RNA-sequencing was performed to explore the association between radiomics and tumor microenvironment. Correlation and variance analyses were conducted to examine changes of radiomics among patients with different therapeutic responses and after neoadjuvant chemotherapy. The association and quantitative relation of radiomics and epigenetic molecular characteristics were further analyzed to reveal the mechanisms of radiomics.

**Results:**

The RDeepNet model showed a significant association with recurrence-free survival (RFS) (HR 0.03, 95% CI 0.02–0.06, *P* < 0.001) and achieved AUCs of 0.98, 0.94, and 0.92 for 1-, 2-, and 3-year RFS, respectively. In the validation and testing cohorts, the RDeepNet model could also clarify patients into high- and low-risk groups, and demonstrated AUCs of 0.91 and 0.94 for 3-year RFS, respectively. Radiomic features displayed differential expression between the two risk groups. Furthermore, the generalizability of RDeepNet model was confirmed across different molecular subtypes and patient populations with different therapy regimens (All* P* < 0.001). The study also identified variations in radiomic features among patients with diverse therapeutic responses and after neoadjuvant chemotherapy. Importantly, a significant correlation between radiomics and long non-coding RNAs (lncRNAs) was discovered. A key lncRNA was found to be noninvasively quantified by a deep learning-based radiomics prediction model with AUCs of 0.79 in the training cohort and 0.77 in the testing cohort.

**Conclusions:**

This study demonstrates that machine learning radiomics of MRI can effectively predict RFS after surgery in patients with breast cancer, and highlights the feasibility of non-invasive quantification of lncRNAs using radiomics, which indicates the potential of radiomics in guiding treatment decisions.

**Supplementary Information:**

The online version contains supplementary material available at 10.1186/s13058-023-01688-3.

## Background

Breast cancer is a leading cause of cancer-related mortality in women worldwide, with recurrence rates of 10–15% within 5 years of diagnosis [1, 2]. Currently, the 70-gene expression profile [3] and 21-gene recurrence score assays [4] are recommended in clinical practice to predict the risk of recurrence and guide decisions regarding adjuvant chemotherapy [5]. However, the high cost of these assays and limited availability of tissue samples for assessment pose challenges to their widespread adoption, potentially overlooking the spatial heterogeneity of breast tumors. Furthermore, these options are only suitable for luminal subtype patients, leaving non-luminal subtype patients at risk of over or undertreatment. In current clinical practice, patients with hormone receptor (HR)-positive or human epidermal growth factor receptor 2 (HER2)-positive tumors receive endocrine therapy or HER2-targeted therapy, respectively. However, there is considerable variation in survival rates among patients within the same treatment strategy. Therefore, a more universally applicable and accurate method is needed to identify patients at high or low risk of recurrence, facilitating personalized treatment decisions and achieving precision therapy.

In recent years, deep learning methods, in particular convolutional neural networks, have become widely used for analyzing nonstructural image data and have demonstrated their effectiveness in capturing image features [6]. For instance, a previous study proposed a multi-task deep learning approach for segmenting tumors and predicting treatment response based on magnetic resonance imaging (MRI) scans of rectal cancer patients [7]. Moreover, in the field of survival analysis, a deep learning survival neural network (DeepSurv) has been developed, which combines the Cox proportional hazards model with deep learning techniques [8]. These studies indicated that incorporating the techniques into the field of radiomics could lead to significant advancements in personalized medicine. This study also demonstrated that DeepSurv has the potential to provide treatment recommendations that lead to improved survival outcomes.

Although radiomic features have been widely utilized for predicting outcomes in cancer patients, the underlying biological mechanisms are still not well-understood. A recent study demonstrated that radiomic features differ between treated and untreated tumors [9], suggesting that these features may reflect changes in the tumor microenvironment. Consequently, it is imperative to investigate the relationship between radiomic features and therapeutic response. Additionally, there is a growing research interest in the epigenetic changes that occur in cancer, with long non-coding RNAs (lncRNAs) gaining recognition for their clinical value. However, the detection methods for lncRNAs currently limit their clinical application. A previous study proposed an artificial intelligence system that employed CT images to predict the epidermal growth factor receptor (EGFR) genotype and prognosis with EGFR-tyrosine kinase inhibitors [10], which reminds us the potential for quantifying lncRNA expression using radiomics. Due to the association between radiomic features and therapeutic response or epigenetics remains uncertain, and prior findings lack robust validation, it certainly seems worthwhile to explore the possible biological basis of radiomics and develop noninvasive tools for detecting lncRNA expression.

In this multicenter study, we constructed the interpretable deep-learning-based Radiomic DeepSurv Net (RDeepNet) model to predict recurrence risk, and evaluated the changes in radiomics before and after therapy with consideration of the therapy response status. The association between radiomic features and lncRNAs was further assessed to explore the potential epigenetic biological underpinning of nonmetastatic invasive breast cancer.

## Methods

### Study design and patients

This study was conducted in accordance with the STROBE guideline checklist [11]. This study included three phases to train and validate the RDeepNet model for prediction of recurrence-free survival (RFS) and explore the association between radiomics and the treatment or epigenetic biological underpinning. In the RDeepNet model construction and validation phase (phase 1), the RDeepNet model was constructed with a combination of the intra- and peritumoral radiomic features using contrast-enhanced T1-weighted imaging (T1 + C) and T2-weighted imaging (T2WI) sequences, which aimed to pinpoint patients with a high or low risk of recurrence. The RDeepNet model was validated in an independent external validation cohort and a testing cohort. RNA-sequencing (RNA-seq) was performed to preliminarily explore the potential molecular mechanisms of radiomics. In phase 2, correlation and variance analyses were conducted to examine the changes of radiomics in patients before and after neoadjuvant chemotherapy with the response status. Based on the above findings, the association and quantitative relation of radiomics and epigenetic molecular characteristics were further analyzed with RNA-seq data in phase 3.

A total of 1,186 nonmetastatic invasive breast cancer patients were retrospectively recruited from four institutions in China, of which 73 patients did not pass the quality control (55 patients were not histologically confirmed to have stage I–III invasive breast cancer [12], and 18 patients lacked an MRI before surgery), and 1113 patients were finally enrolled. A total of 698 patients recruited from the national hospitals Sun Yat-sen Memorial Hospital of Sun Yat-sen University (Guangzhou, China) and Sun Yat-sen University Cancer center (Guangzhou, China) between March 23, 2011, and August 26, 2019, were assigned to a training cohort. Then, 171 patient cases collected from the Shunde Hospital of Southern Medical University (Foshan, China) and the Tungwah Hospital of Sun Yat-sen University (Dongguan, China) between March 09, 2012, and September 21, 2019, were used as the validation cohort. A total of 244 patients from the Sun Yat-sen Memorial Hospital of Sun Yat-sen University (Guangzhou, China) between April 19, 2013, and December 05, 2018, were assigned to the testing cohort. We retrospectively collected 92 formalin-fixed paraffin-embedded (FFPE) biopsy tissues from patients treated at the Sun Yat-sen Memorial Hospital of Sun Yat-sen University. All samples were reassessed by two pathologists and were found to contain more than 70% tumor cells. A total of 72 patients, who had both T1 + C and T2WI sequences from The Cancer Genome Atlas (TCGA) and The Cancer Imaging Archive (TCIA), were assigned to the TCGA cohort for assessing the efficacy of the deep learning prediction model.

The inclusion criteria were female patients aged at least 18 years with histological confirmation of stage I–III invasive breast cancer [12], underwent breast tumor and axillary MRI scans before surgery and axillary lymph node dissection, and who experienced perioperative therapy. Cases of patients with other previous or simultaneous tumors, incomplete pathological information, or unavailable standard MRI scans with or without contrast enhancement were excluded. The outcome was RFS, calculated from the date of surgery until the date of the most recent medical review or diagnosis of recurrence, or metastasis, and the association of radiomics with lncRNAs.

The four molecular subtypes of breast tumors were defined according to the St. Gallen Consensus Conference 2013 [13], with biomarkers measured by immunohistochemistry or in situ hybridization. Luminal A subtype patients were defined as estrogen receptor (ER)- and progesterone receptor (PR)-positive, HER2-negative, and Ki-67 level < 14%. Luminal B subtype patients were defined as ER-positive and over-expressed/amplified HER2, or ER-positive and HER2-negative, with Ki-67 level > 14%, or PR-negative/low. In contrast, ER- and PR-negative, HER2-positive subtype patients had over-expressed/amplified HER2, and triple-negative breast cancer (TNBC) subtype patients were HER2-negative.

### Procedures of transcriptome RNA sequencing

Total RNA was extracted from FFPE samples using the QIAGEN FFPE RNeasy kit (QIAGEN GmbH, Hilden, Germany). RNA was analyzed using an Agilent RNA 6000 Nano Kit (Aglient Technologies, Santa Clara, CA, USA), and RNA integrity numbers were determined to evaluate RNA integration using an Agilent Bioanalyzer 2100 (Aglient Technologies, Santa Clara, CA, USA). An input of 500 ng of total RNA was amplified using an Ovation FFPE WTA System (NuGEN, San Carlos, CA, USA), and a NEBNext® Ultra™ II DNA Library Prep Kit (Illumina) was used for fragmentation and labeling. The quality and quantity of amplified libraries were evaluated using Qubit (Invitrogen, Carlsbad, CA, USA) and Agilent Bioanalyzer 2100 (Aglient Technologies, Santa Clara, CA, USA). All libraries were sequenced using a DNBSEQ-T7RS (MGI) with 100 bp paired-end reads. Base call files were converted to the fastq format using cal2Fastq. Raw data were normalized using the fastp (version 0.20.1) for data processing.

### Radiomic feature extraction

The acquisition protocol of the multiparametric MRI (including T1 + C, and T2WI) used across all institutions and the MR scanner parameters are described in Additional file 1: eAppendix 1 and Additional file 1: Table S1. All of the MRIs were normalized to obtain a standard normal distribution of image intensities using the N4ITK Bias Correction code. The 3D regions of interest (ROIs) in the breast intratumoral area and the peritumoral area (10-mm extension outward of the tumor parenchyma) were semi-automatically segmented using the 3D Slicer software (https://www.slicer.org/, version 4.10.2) [14]. The 3D regions of intra- and peritumoral (DICOM format) were transferred to the SlicerRadiomics code, a texture extraction platform based on the python package “PyRadiomics” [15]. For each patient, 3,452 quantitative radiomic features (863 features from each ROI in each sequence, including 12 diagnostic features, 107 original features, and 744 wavelet features) were extracted to analyze shape, size, intensity, morphology, and texture. Besides diagnostic features, the remaining radiomic features were categorized into seven groups: shape descriptors, first-order statistics, gray-level co-occurrence matrix (GLCM), gray-level size zone matrix (GLSZM), gray-level run-length matrix (GLRLM), gray-level dependence matrix (GLDM), and neighboring gray tone difference matrix (NGTDM). More details regarding the radiomic feature extraction are described in Additional file 1: eAppendix 2.

### RDeepNet model building and validation

The Cox proportional hazards deep neural network, DeepSurv [8], was applied to construct the RDeepNet model for predicting individual recurrence risk. The network took 3,452 radiomic features as input for each patient. For the recurrence risk, the RDeepNet score was calculated with a single output node based on the negative log-partial likelihood function. The RFS predicted from the RDeepNet model was then assessed in the validation cohort and the testing cohort, respectively. More details about the network were described previously [8].

### Radiomic features varied among patients with different responses and after neoadjuvant chemotherapy

In total, 127 (52%) of the 244 patients from the testing cohort had radiomic features from before and after neoadjuvant chemotherapy, of which 72 (57%) patients were evaluated as responsive (complete response + partial response) to the therapeutic, with the standard of Response Evaluation Criteria in Solis Tumors (RECIST). The other 55 (43%) patients were defined as unresponsive (stable disease + progressive disease). The differential therapy-related radiomic features between responsive and unresponsive patients or before and after neoadjuvant chemotherapy were identified using the limma package, *t* test and paired samples *t* test, respectively. The heatmaps of the differentially expressed radiomic features were obtained with the R package pheatmap. The correlation matrix maps of the radiomic features extracted from intratumoral region were performed with the R package ggplots and RColorBrewer.

### Exploration of the molecular mechanisms of radiomics

To explore the related biological mechanisms of radiomics, we performed RNA-seq for 92 patients from the training cohort. Additional file 1: Table S2 shows the clinicopathological characteristics of these patients. The compared files were downloaded from https://www.ensembl.org/index.html and annotated with Perl software according to the ensemble ID of sequencing results. Next, the gene length was compared through the Gencode27 database on the basis of the counts data. Then, the counts data were converted into TPM data, and the lncRNAs were distinguished in accordance with the Ensembl database.

The *t* test and limma package were used to identify differentially expressed genes between high- and low-risk patients according to the RDeepNet score. Then, the proportion of the tumor immune microenvironment were quantified in the 92 patients with the ssGSEA algorithm, which were used for highly sensitive and specific discrimination of 28 human immune cell phenotypes, including B cells, T cells, natural killer cells, macrophages, dendritic cells, and myeloid subsets. Spearman’s rank correlation analysis and limma package were used between high- and low-risk patients to further explore the association between radiomics and the tumor immune microenvironment.

To explore the potential epigenetic biological underpinning of radiomics, 15 lncRNAs were selected using the Spearman’s rank correlation analysis and univariable Cox proportional hazards regression model in 92 patients with RNA-seq data. The limma package was utilized to identify the differential radiomic features between patients with high and low expression of the key lncRNA. The Gene Ontology (GO) and Kyoto Encyclopedia of Genes and Genomes (KEGG) analyses were performed using the clusterProfiler R package [16]. The pathways were also identified by running a gene set variation analysis (GSVA) with the R package gsva. The pathway enrichment analyses were considered statistically significant, with *P* values and false discovery rates of less than 0.05. Next, the deep learning prediction model of lncRNA expression was built with the intratumoral radiomic features based on the multilayer neural network (MLP) [17, 18]. A total of 92 patients with RNA-seq data were included for training the model, and 72 patients with both T1 + C and T2WI sequences from TCGA and TCIA were assigned to the TCGA cohort for assessing the efficacy of the model.

### Statistical analysis

Fisher’s exact tests were performed to examine differences in the occurrence of categorical variables, while independent *t* tests were used to compare differences in continuous variables between the two groups. Survival was calculated using the Kaplan–Meier method and the log-rank test. Hazard ratios (HRs) and 95% confidence intervals (Cls) were calculated using a Cox regression analysis. Patients were categorized into high and low-risk groups with the optimal cutoff values defined by the R package ggsurvimier. The prognostic or predictive accuracy of the RDeepNet model and prediction model of lncRNA expression was assessed by using receiver operating characteristic curve (ROC) analysis. The performance of the RDeepNet model for RFS prediction and prediction model of lncRNA expression was evaluated by assessing sensitivity and specificity calculated by using the area under the ROC curve (AUC) method. For all analyses, two-sided *P*-values less than 0.05 were considered statistically significant. Statistical analyses were performed using R software (version 4.0.0).

## Results

### Patient characteristics

**Fig. 1:**
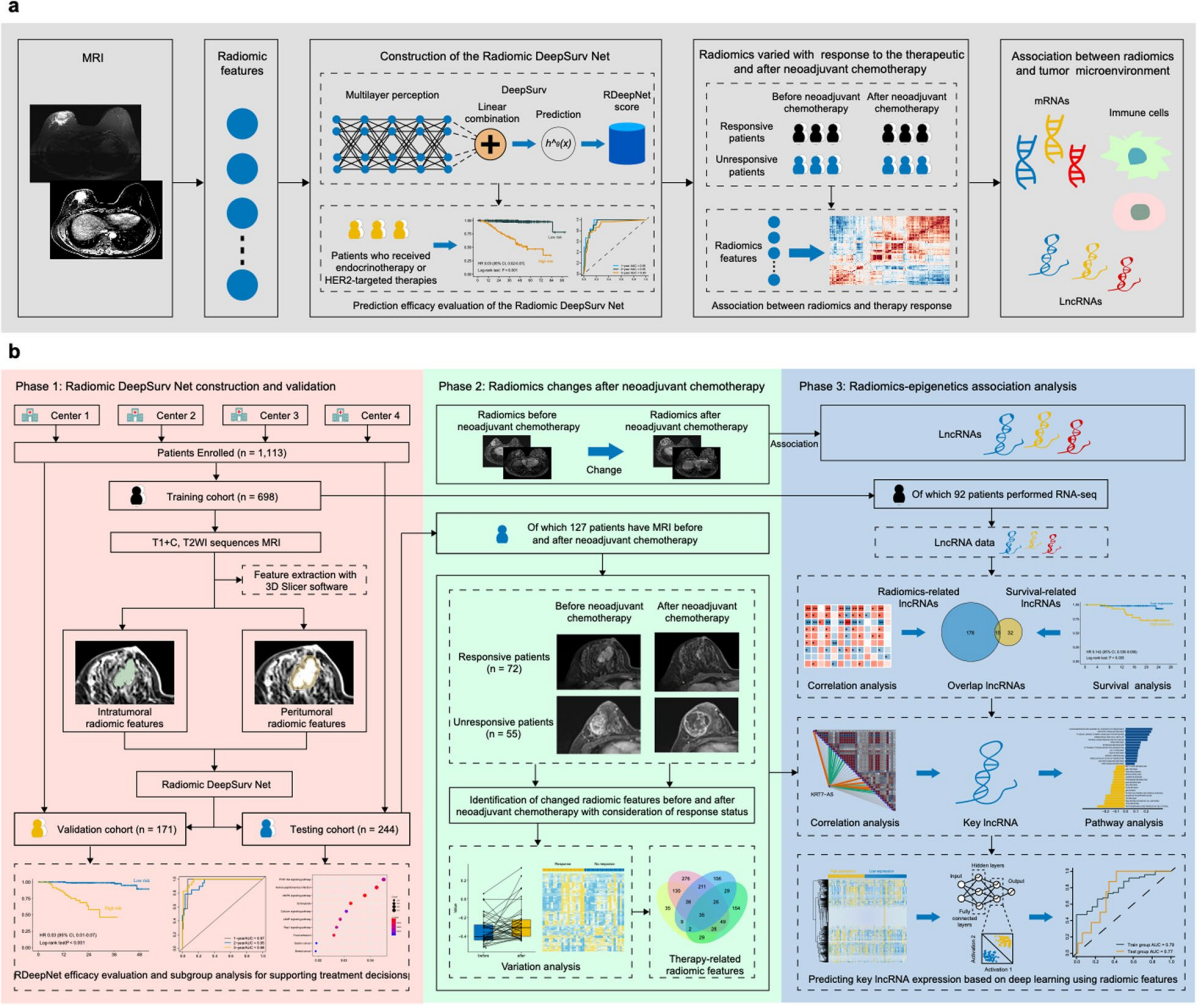
Patient recruitment and study design. The deep-learning-based Radiomic DeepSurv Net was constructed with MRI radiomic features, and was found to be employed for RFS prediction and associated with therapy response and tumor microenvironment (**a**). This study included three phases to train and validate the RDeepNet model for prediction of RFS and explore the association between radiomics and the treatment or epigenetic biological underpinning. In phase 1, a total of 1113 patients with preoperative MRI from four institutions were enrolled in this study to construct and validate the RDeepNet model for the prediction of recurrence risk. In phase 2, correlation and variance analyses were conducted to examine the change in radiomics in patients before and after neoadjuvant chemotherapy with the response status. In phase 3, 92 of 698 patients from the training cohort underwent RNA-seq with the FFPE samples to obtain lncRNAs data and analyze the association between radiomics with lncRNAs and RFS (**b**). LncRNAs, long non-coding RNAs; MRI, Magnetic resonance imaging; RFS, recurrence-free survival; T1 + C, contrast-enhanced T1-weighted imaging; T2WI, T2-weighted imaging

This study included three phases to train and validate the RDeepNet model for prediction of RFS and explore the association between radiomics and the treatment or epigenetic biological underpinning, and we eventually achieved the prediction for expression of lncRNA with radiomic features based on deep learning. A total of 1113 patients from four academic institutions in China were eligible for this study (Additional file 1: Table S3). Additional file 1: Table S4 shows the clinicopathological characteristics of patients in the training cohort (*n* = 698), the validation cohort (*n* = 171), and the testing cohort (*n* = 244). Endocrine therapy was administered to 446 (64%) of 698 patients in the training cohort, 103 (60%) of 171 patients in the validation cohort, and 135 (55%) of 244 patients in the testing cohort. HER2-targeted therapy was administered to 210 (30%) of 698 patients in the training cohort, 50 (29%) of 171 patients in the validation cohort, and 93 (38%) of 244 patients in the testing cohort. From the testing cohort, 244 patients underwent neoadjuvant chemotherapy. The median follow-up time was 44.7 months (IQR 34.0–57.3) for the training cohort, 40.4 months (IQR 29.3–62.3) for the validation cohort, and 39.9 months (IQR 36.1–50.9) for the testing cohort. The 3-year RFS rate was 93.6% (95% CI 91.7–95.5%) for the training cohort, 96.7% (95% CI 93.8–99.6%) for the validation cohort, and 93.3% (95% CI 90.0–96.6%) for the testing cohort. Detailed information regarding the patient recruitment and study design is described in Fig. 1.

### RDeepNet model for recurrence risk prediction and supporting treatment decisions

**Fig. 2:**
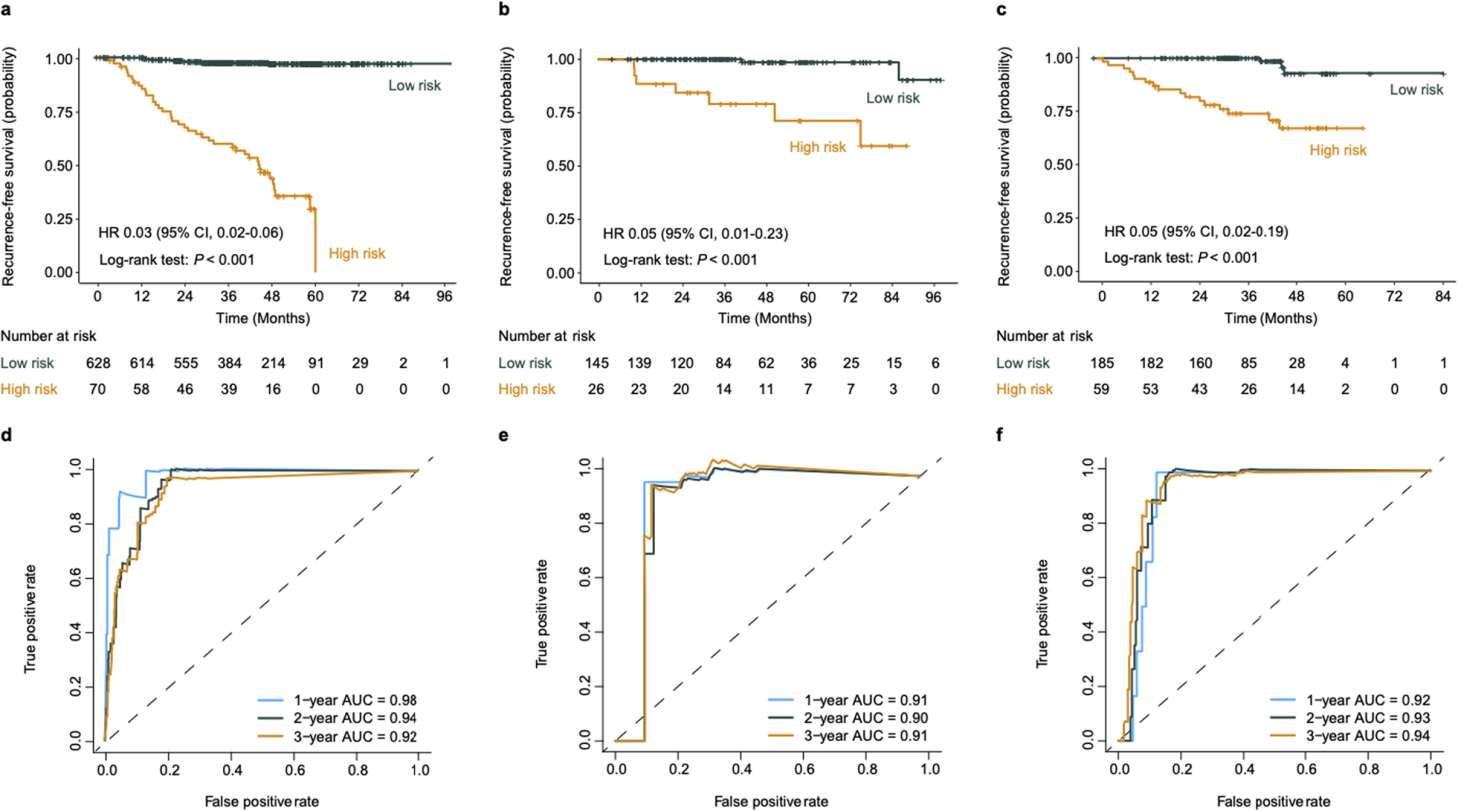
Performance of the RDeepNet model for predicting the recurrence risk in the training, validation, and testing cohorts. Kaplan–Meier curves of RFS according to the RDeepNet model in the **a** training cohort, **b** validation cohort, and **c** testing cohort. ROC curves and 1-, 2-, 3-year AUCs were used to assess the prognostic accuracy of the RDeepNet model in the **d** training cohort, **e** validation cohort, and **f** testing cohort. *P* values were calculated using the unadjusted log-rank test, and hazard ratios were calculated by a univariate Cox regression analysis. AUC, area under the receiver operating characteristics curve; CI, confidence interval; HR, hazard ratio; RFS, recurrence-free survival; ROC, receiver operating characteristic

The RDeepNet model, combining both intratumoral and peritumoral radiomic features, was developed. The RDeepNet model categorized patients into high- and low-risk groups with an optimal cutoff value (1.10). The RDeepNet model assigned 70 (10%) of 698 patients to the high-risk group, and there were significant differences in RFS between the high- and low-risk groups (HR 0.03, 95% CI 0.02–0.06, *P* < 0.001). In the validation cohort, 26 (15%) of the 171 patients were assigned to the high-risk group, which had shorter RFS (HR 0.05, 95% CI 0.01–0.23, *P* < 0.001). In the testing cohort, 59 (24%) of the 244 patients with high risk had shorter RFS (HR 0.05, 95% CI 0.02–0.19, *P* < 0.001) (Fig. 2a–c). Moreover, the RDeepNet model showed AUCs for the 1-, 2-, and 3-year RFS of 0.98, 0.94, and 0.92, respectively, in the training cohort; 0.91, 0.90, and 0.91, respectively, in the validation cohort; and 0.92, 0.93, and 0.94, respectively, in the testing cohort (Fig. 2d–f).

**Fig. 3:**
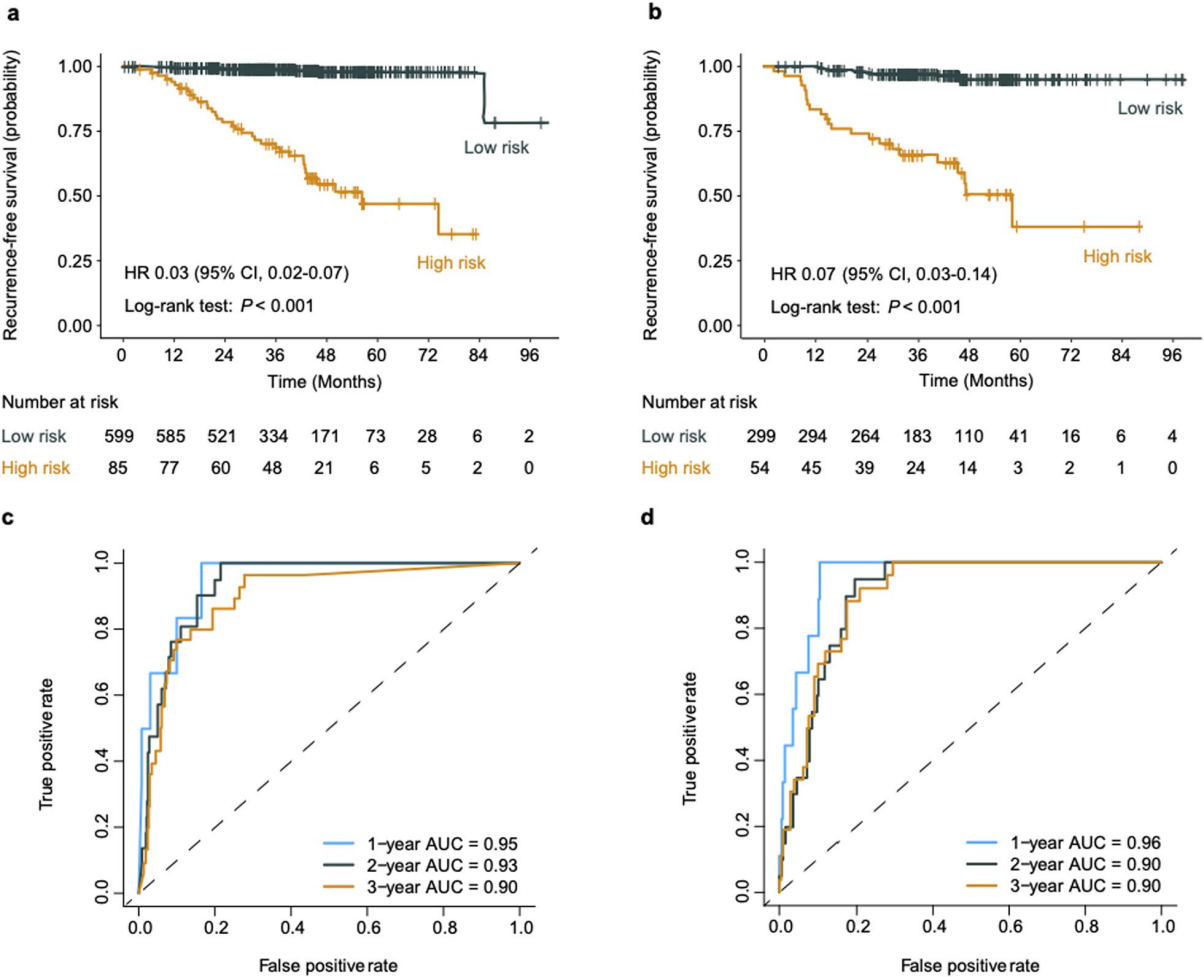
Performance of the RDeepNet model for recurrence risk prediction in patients with different therapy regimens. Kaplan–Meier curves of RFS according to the RDeepNet model in the subgroups of patients with **a** endocrine therapy and **b** HER2-targeted therapy. ROC curves and 1-, 2-, 3-year AUCs were used to assess the prognostic accuracy of the RDeepNet model in the subgroups of patients with **c** endocrine therapy and **d** HER2-targeted therapy. *P* values were calculated using the unadjusted log-rank test, and hazard ratios were calculated by a univariate Cox regression analysis. AUC, area under the receiver operating characteristics curve; CI, confidence interval; HR, hazard ratio; HER2, human epidermal growth factor receptor 2; RFS, recurrence-free survival; ROC, receiver operating characteristic

In addition, the RDeepNet model was employed to classify a high and low risk of recurrence in patients by considering the molecular subtypes of cancer. Encouragingly, the RDeepNet model could discriminate high- from low-risk patients in the subgroups of luminal A (*P* < 0.001), luminal B (HR 0.06, 95% CI 0.03–0.10, *P* < 0.001), HER2-positive (HR 0.05, 95% CI 0.01–0.22, *P* < 0.001), and TNBC (*P* < 0.001) patients (Additional file 1: Fig. S1). Moreover, the RDeepNet model could recognize high- and low-risk patients among patients treated with endocrine therapy (HR 0.03, 95% CI 0.02–0.07, *P* < 0.001) and patients treated with HER2-targeted therapy (HR 0.07, 95% CI 0.03–0.14, *P* < 0.001) (Fig. 3a, b). In parallel, the efficacy of the RDeepNet model showed AUCs of 0.95, 0.93, and 0.90 for 1-, 2-, 3-year RFS prediction among patients treated with endocrine therapy. These AUCs were 0.96, 0.90, and 0.90, respectively, among patients treated with HER2-targeted therapy (Fig. 3c, d).

According to the RDeepNet model, the radiomic features were expressed differentially between the high- and low-risk groups among patients in the training cohort (Additional file 1: Fig. S2). To determine the potential mechanisms of radiomics, RNA-seq for 92 patients from the training cohort was performed. We identified 148 differentially expressed genes between the high- and low-risk groups (Additional file 1: Fig. S3a). The pathway enrichment analyses showed that these genes were highly enriched in PI3K-Akt signaling pathway, MAPK signaling pathway, and cell-migration-related genomic biological processes (Additional file 1: Fig. S3b, c). These genes were also involved in various pathways as well as physiological and pathological processes, which were associated with tumor, immunity and metabolism, such as JAK STAT signaling pathway, cytokine interaction, and the energy metabolism (Additional file 1: Fig. S3d). We further evaluated the association between the RDeepNet score and immune cells (Additional file 1: Fig. S4a). Correlation analysis indicated that the RDeepNet score was significantly related to activated dendritic cells, CD56bright natural killer cells, central memory CD4 T cells, effector memory CD4 T cells, effector memory CD8 T cells, myeloid-derived suppressor cell, T follicular helper cells, Type 1 T helper cells, Type 17 T helper cells, and Type 2 T helper cells (Additional file 1: Fig. S4b). Furthermore, variation analysis of immune cells showed that patients from the high-risk group had lower expression of CD56dim natural killer cells and central memory CD8 T cells but higher expression of effector memory CD8 T cells compared with low-risk patients (Additional file 1: Fig. S4c).

### Radiomic features varied from different therapy responses and post-neoadjuvant chemotherapy

**Fig. 4:**
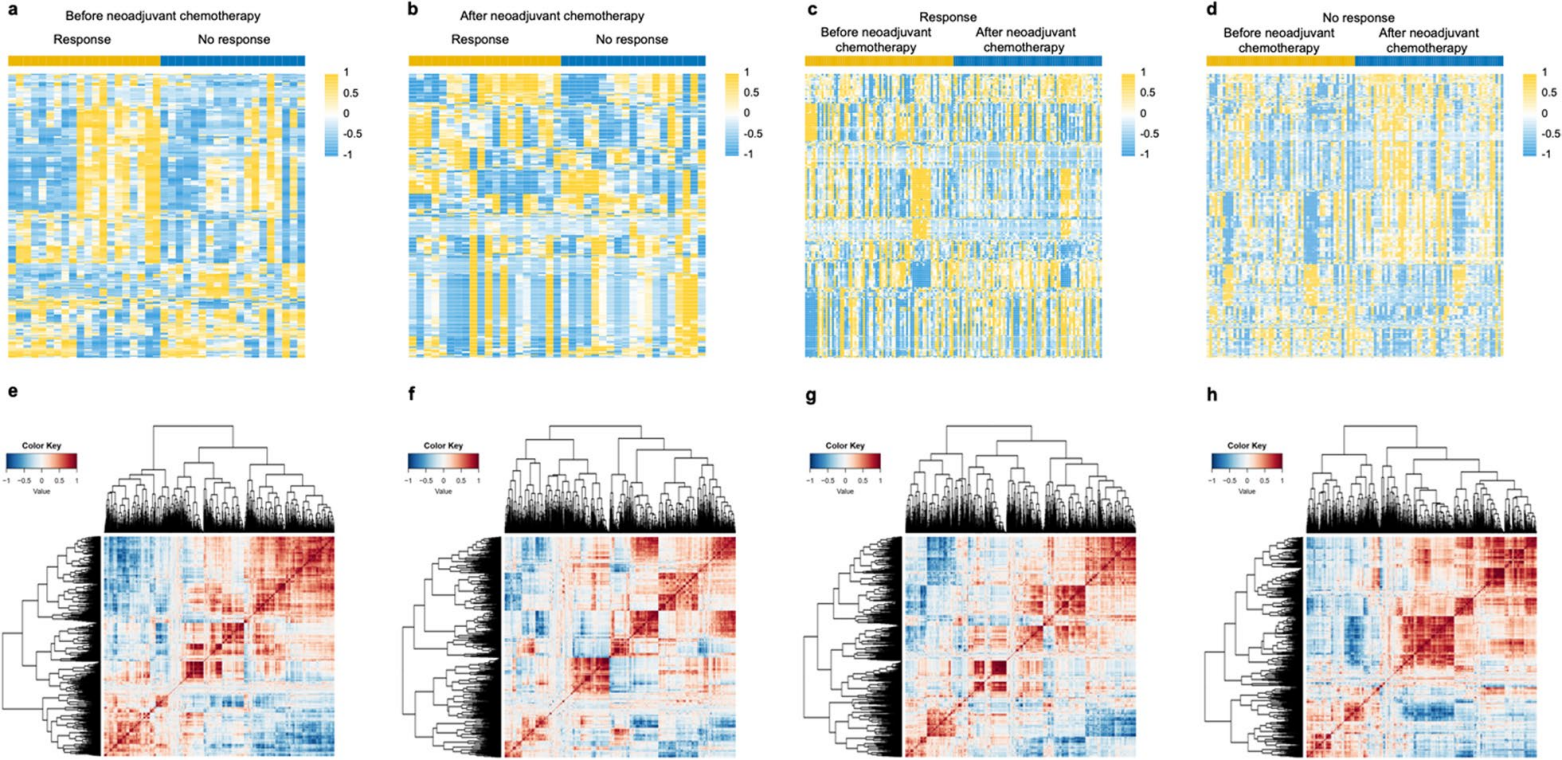
Radiomic feature maps between patients with different therapeutic responses before and after neoadjuvant chemotherapy. Heatmaps of the differential radiomic features between responsive patients and unresponsive patients **a** before neoadjuvant chemotherapy and **b** after neoadjuvant chemotherapy. Heatmaps of the differential radiomic features between before neoadjuvant chemotherapy and after neoadjuvant chemotherapy in **c** responsive patients and **d** unresponsive patients. Correlation matrix maps of the radiomic features generated from patients with response to the treatment **e** before neoadjuvant chemotherapy and **f** after neoadjuvant chemotherapy, and patients with no response to the treatment **g** before neoadjuvant chemotherapy and **h** after neoadjuvant chemotherapy. *P* values were calculated using the unadjusted log-rank test and paired samples *t* test

After the RDeepNet model construction and validation phase, in phase 2, we aimed to determine whether radiomic features can predict changes after neoadjuvant chemotherapy. Radiomic variation and correlation analyses were performed on 127 patients (including 72 responsive patients and 55 unresponsive patients) who had intratumoral radiomic features, both before and after neoadjuvant chemotherapy. A total of 1726 intratumoral radiomic features were analyzed with consideration of patients’ response to the therapy. Before neoadjuvant chemotherapy, 456 radiomic features were found to be differentially expressed between responsive and unresponsive patients (Fig. 4a). It was observed that there were 352 variant radiomic features between the above two groups of patients after neoadjuvant chemotherapy (Fig. 4b). In addition, 306 and 793 radiomic features were found to be statistically different after the neoadjuvant chemotherapy in the responsive and unresponsive patients, respectively (Fig. 4c, d). The correlation between these features changed obviously after neoadjuvant chemotherapy. Patients after neoadjuvant chemotherapy had higher correlations among some radiomic features than patients before neoadjuvant chemotherapy (Fig. 4e–h). We further took the overlaps of the above differential radiomic features, and 35 radiomic features (therapy-related features) were considered to be the key features that were primarily correlated with therapy (Additional file 1: Fig. S5). It is worth noting that 27 of the 35 radiomic features were found to be significantly different between the high- and low-risk groups. Most of the key differential radiomic features were found to belong to the classification of the GLCM or GLRLM. More details about the classification of these features are shown in Additional file 1: Table S5.

### The association and quantitative relation between radiomics and LncRNAs

**Fig. 5:**
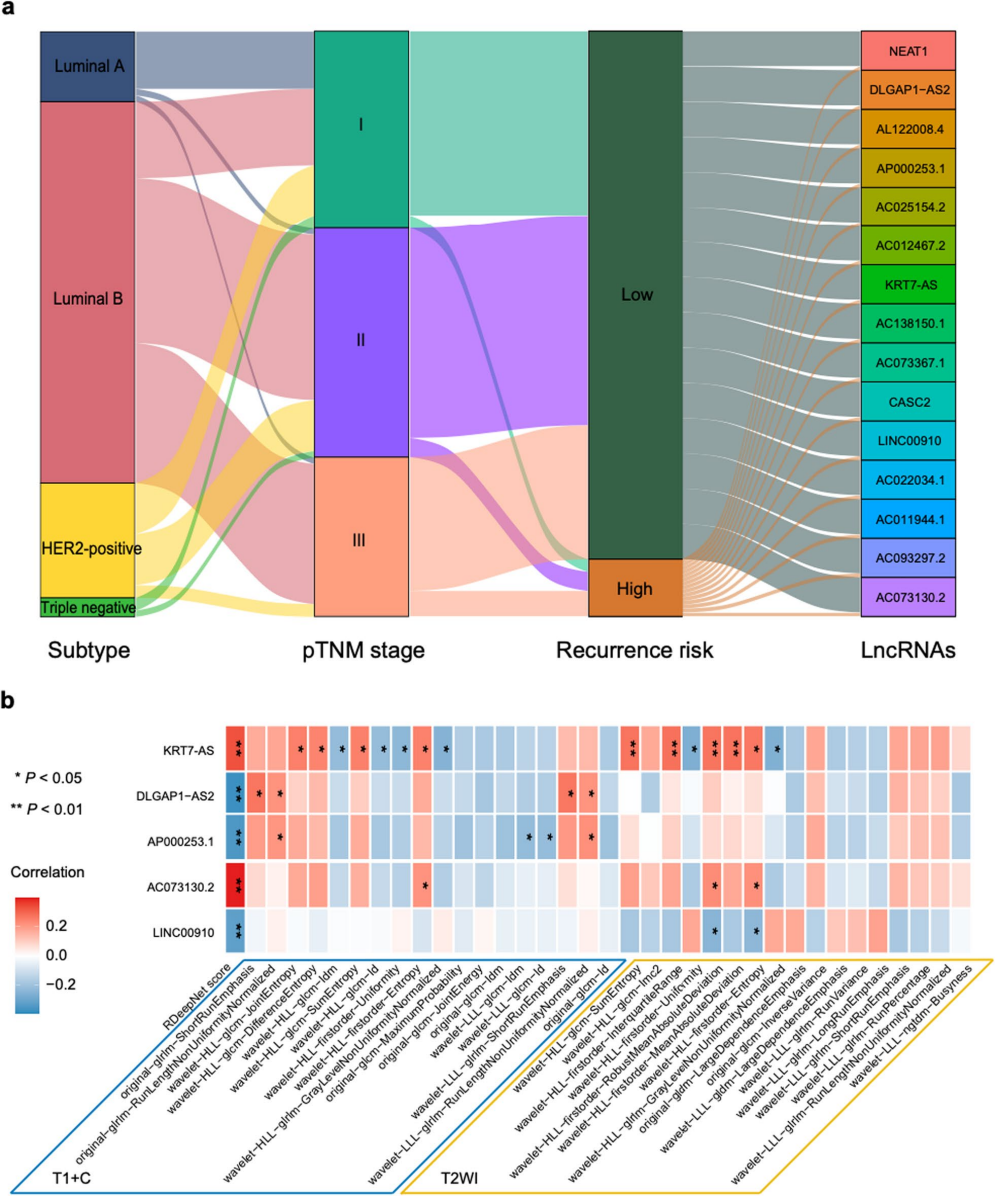
Correlation of radiomics with lncRNAs. **a** Individual molecular subtype, pTNM stage and lncRNAs were associated with high and low recurrence risk according to the RDeepNet model. **b** Correlation matrix of the therapy-related radiomic features with lncRNAs. *P* values were calculated using the unadjusted log-rank test; rho values were calculated by Spearman rank correlation analysis. HER2, human epidermal growth factor receptor 2; LncRNAs, long non-coding RNAs; pTNM = pathological tumor–node–metastasis stage; T1 + C, contrast-enhanced T1-weighted imaging; T2WI, T2-weighted imaging

Based on the above findings, the association of radiomics and epigenetic molecular characteristics was explored in phase 3 based on the results of RNA-seq. A total of 12,312 lncRNAs were produced from the transcriptome sequencing data for each patient. To look for the crucial lncRNAs, correlation analysis between lncRNAs and the RDeepNet score was performed, which allowed the identification of 47 lncRNAs at a significance threshold of *P* < 0.05. Of these, 15 were associated significantly with RFS (All *P* < 0.05) (Additional file 1: Figs. S6–8). The full list of the 15 lncRNAs is detailed in Additional file 1: Table S6. The association among the risk stratification by the RDeepNet model, pathological tumor-node-metastasis (pTNM) stage, molecular subtypes, and the 15 lncRNAs is visualized in Fig. 5a. Five (KRT7-AS, DLGAP1-AS2, AP000253.1, AC073130.2, LINC00910) of 15 lncRNAs were identified to be highly correlated with some of the above 35 therapy-related radiomic features (Fig. 5b).

**Fig. 6:**
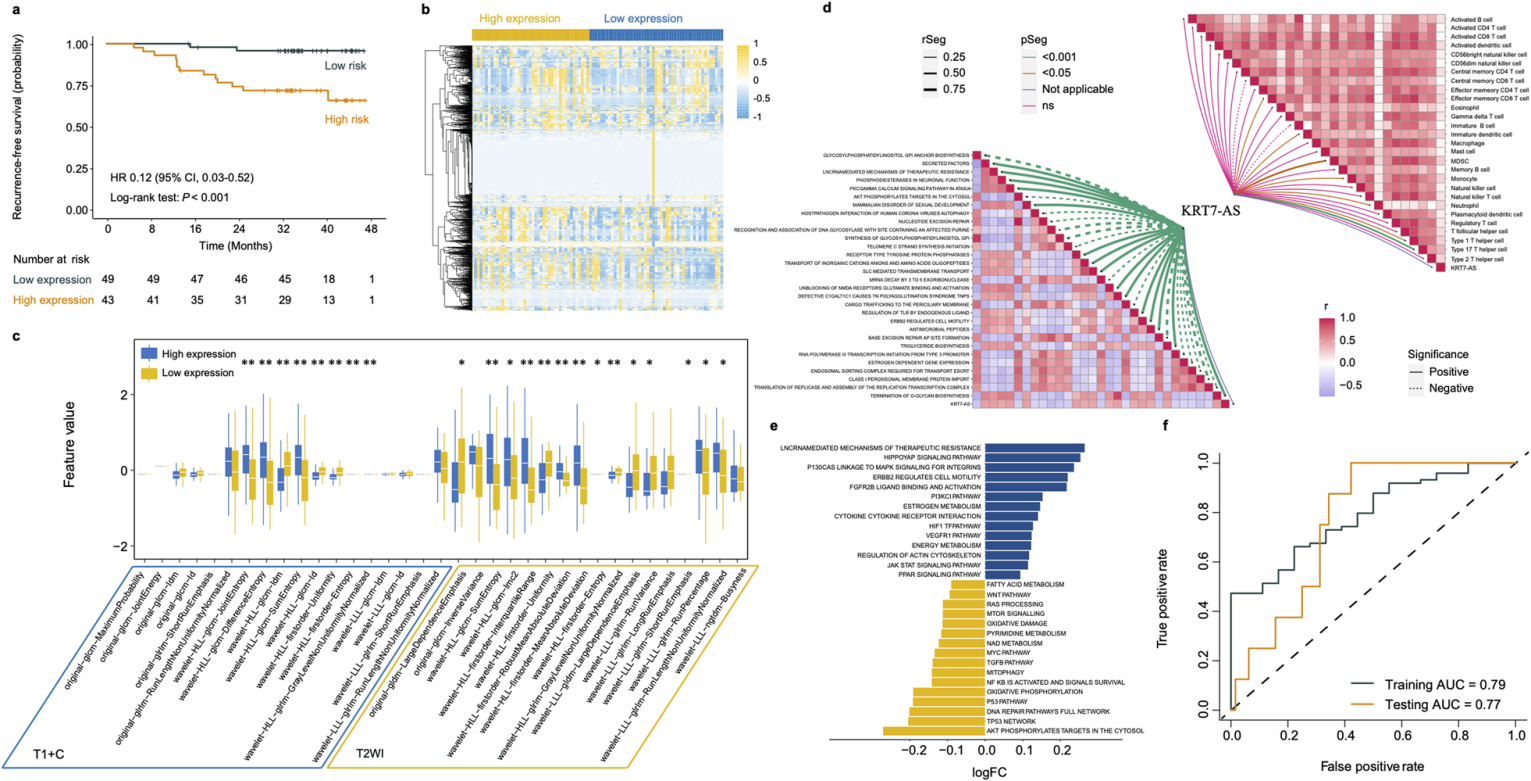
Association of lncRNA KRT7-AS with RFS and radiomics. **a** Kaplan–Meier curves of RFS according to the expression of lncRNA KRT7-AS. **b** Overall distribution and **c** differential expression of the radiomic features from T1 + C and T2WI sequences in patients with high and low expression of lncRNA KRT7-AS, *P < 0.05, **P < 0.01. **d** The lncRNA KRT7-AS-related pathways and immune cells. **e** The GSVA pathway enrichment analysis of lncRNA KRT7-AS-based genes*.* **f** ROC curves and AUCs were used to assess the accuracy of the deep learning model for predicting lncRNA KRT7-AS expression.* P* values were calculated using the unadjusted log-rank test, and hazard ratios were calculated by a univariate Cox regression analysis. AUC, area under the receiver operating characteristics curve; CI, confidence interval; GSVA, gene set variation analysis; HR, hazard ratio; LncRNA, long non-coding RNA; ROC, receiver operating characteristic; T1 + C, contrast-enhanced T1-weighted imaging; T2WI, T2-weighted imaging

The lncRNA KRT7-AS in particular was observed to be associated with RFS (HR 0.12, 95% CI 0.030–0.52, *P* < 0.001) (Fig. 6a), and was correlated linearly with most of the therapy-related radiomic features. Similarly, 22 of 35 therapy-related radiomic features were found to be differentially expressed in patients with differential expression of lncRNA KRT7-AS (Fig. 6b, c). To explore the potential biological underpinning, pathway enrichment analysis was conducted to evaluate the enrichment of the lncRNA KRT7-AS-related and survival-based genes. Figure 6d shows the lncRNA KRT7-AS mainly associated with various tumor- or metastasis-associated pathways and processes, such as the Akt phosphorylates, nucleotide excision repair, and ERBB2 regulates cell motility. The lncRNA KRT7-AS was also found to be correlated with effector memory CD8 T cells, immature dendritic cells, myeloid-derived suppressor cells, monocytes, neutrophils, type 1 T helper cells, and type 17 T helper cells. The results show that genes based on different expression of KRT7-AS were involved in the process of lncRNA-mediated mechanisms of therapeutic resistance, the Hippo-YAP signaling pathway, and TP53 network, which was also associated with tumors and survival (Fig. 6e). These results are consistent with previous research findings that the lncRNA KRT7-AS could promote tumor progression [19–21].

The above findings show that the lncRNA KRT7-AS was obviously associated with radiomics and mediated in the progression of breast cancer. They remind us that it was feasible to predict the expression of KRT7-AS using radiomics. A deep learning prediction model was constructed based on the MLP among the 92 patients from the training cohort and tested in 72 patients from TCGA and TCIA. Encouragingly, the prediction model achieved AUC values of 0.79 in the training cohort and 0.77 in the TCGA testing cohort (Fig. 6f). This result reveals the possibility of noninvasive quantification for lncRNAs by deep learning radiomics.

## Discussion

In this multicenter study, deep learning algorithms based on the T1 + C and T2WI sequences combining the intratumoral and peritumoral radiomic features were found to be significantly associated with RFS and presented a higher predictive value for RFS. The RDeepNet model successfully classified patients with different breast cancer molecular subtypes or different therapy regimens in high- and low-recurrence risk categories. Furthermore, it was observed that some radiomic features varied from patients with different response statuses and after neoadjuvant chemotherapy. More importantly, the radiomics showed significant association with lncRNAs according to the results of RNA-seq, and the expression of lncRNA could be quantified by radiomics. Overall, this study developed and validated a prognostic network for individualized prediction of high and low recurrence risk, which serves as an effective tool for survival prediction and clinical decision-making in patients with nonmetastatic invasive breast cancer. Moreover, the potential epigenetic biological underpinning of radiomics was preliminarily revealed, and a non-invasive method was established to predict expression of epigenetic molecule.

While previous studies [22, 23] showed the potential of MRI-based radiomics for predicting breast cancer recurrence, their clinical value was limited because they used a small sample size and single-center cohorts, extracted the radiomic features only from the tumor region, and were based on machine learning algorithms. A previous study [24] constructed a radiomics nomogram based on intratumoral features in 294 invasive breast cancer patients from a single center, and estimated DFS with C-index of 0.76. As far as we know, our study was the first to build a network based on deep learning with both intratumoral and peritumoral radiomic features in multicenter cohorts of more than 1,000 breast cancer patients. Furthermore, we analyzed the efficacy of the RDeepNet model in patients treated with different therapy regimens and the change in radiomics with different therapeutic response or before and after therapy. We also performed RNA-seq to explore the potential epigenetic biological underpinning of radiomics, and achieved noninvasive prediction expression of lncRNA by utilizing radiomic features.

In current clinical practice, patients with positive HR status are considered for endocrine therapy, and HER2-targeted therapy is selected for HER2-positive patients. However, some patients still experience progress owing to therapy resistance [25, 26]. The Oncotype DX21-gene [27] and the PAM50 risk score [28] have been used to predict the response of endocrine therapy, but these methods are invasive and only suitable for a subset of the population. As for HER2-targeted therapy, only HER2 amplification or overexpression predicts an enhanced survival benefit from the HER2-targeted therapy at present. Although a previous study presented an MRI-based signature, which could noninvasively characterize HER2-positive tumor biological factors and estimate the response to HER2-targeted neoadjuvant therapy, the small size sample and highly heterogeneous data limited the application [29]. Therefore, it is urgent to explore other methods for predicting the therapy response in addition to the status of HR or HER2. In this study, the RDeepNet model could recognize recurrence risk among patients treated with endocrine therapy or HER2-targeted therapy, and the efficacy showed all of the AUCs of more than 0.90. These results indicate that the RDeepNet model had the potential to assist in treatment decisions.

In the present study, the differentially expressed genes between the high- and low-risk groups were identified with the RNA-seq data. Results of pathway enrichment analyses show that these genes might be involved in the regulation of host immune responses. The further evaluation demonstrated that the RDeepNet score was significantly related to most immune cells, and high-risk patients showed lower expression of CD56dim natural killer cells. As we know, CD56dim natural killer cells account for more than 90% of natural killer cells and mainly play a cytotoxic role, with stronger killing activity [30]. In addition, the RDeepNet model could identify a high and low risk of recurrence in the testing cohort, in which all of the patients underwent neoadjuvant chemotherapy. It is worth noting that there some radiomic features were differentially expressed before and after neoadjuvant chemotherapy and varied in responsive and unresponsive patients. These radiomic features were defined as therapy-related features. The above findings remind us that radiomics can reflect the change in the tumor microenvironment or molecular characteristics.

In recent years, emerging evidence has suggested that abnormal expression of lncRNAs is a frequent biological phenomenon in tumors and is closely associated with the prognosis of cancer patients. Several studies have indicated that the MRI radiomic profile of cancer patients can predict the prognosis, but the potential biological underpinning of MRI radiomics remains indistinct. We hypothesized that MRI radiomics can reflect the expression of lncRNAs, and therefore provided prognosis information. In this study, based on patients who had both RNA-seq and preoperative MRI data, we screened 15 lncRNAs related to both radiomic features and RFS to confirm our hypothesis. Among these lncRNAs, KRT7-AS was significantly correlated with the therapy-related radiomic features, and the KRT7-AS-based differentially expressed genes were enriched in process of lncRNA-mediated mechanisms of therapeutic resistance and various metastasis- or metabolism-associated pathways. Previous research has found that the increasing stability of lncRNA KRT7-AS could promote breast cancer lung metastasis by regulation of *N*^*6*^-methyladenosine [19]. KRT7-AS also supports gastric cancer and colorectal cancer progression by modulating KRT7 expression [20, 21]. Therefore, the lncRNA KRT7-AS indeed plays an important role in tumor progression, and it is necessary to examine KRT7-AS expression to predict survival.

However, the clinical application of lncRNAs as biomarkers is severely limited owing to the lack of detection methods. Our results suggest that MRI radiomic profiles can help identify potential targets for molecular-based therapy of breast cancer, and MRI examination may be used to monitor the expression level of molecular features during the therapy. Based on the above findings, a deep learning prediction model of KRT7-AS expression was further constructed with MLP and showed high predictive efficacy in both training and testing cohorts. This result can afford non-invasive detection of molecular expression by just acquiring radiomic features, which can assist in conveniently monitoring dynamic changes in tumors. Furthermore, the exploration of the association between lncRNAs and MRI radiomics is just the fundamental starting point, and the potential biological relationship of MRI radiomic profiles with other molecular species, such as DNA methylation, DNA copy number and sequence variation, should be evaluated in the future.

Several limitations existed in the present study. Heterogeneity among the MRI scans from multiple clinical centers was inevitable. The median follow-up was about 40 months. Therefore, the outcomes were limited, and the RDeepNet model could not be applied to predict overall survival. It is necessary to evaluate the radiomic changes with the extension of follow-up time. Due to the relatively low incidence of TNBC among breast cancer patients and the retrospective approach taken in this study, TNBC patients may be under-representation. Previous studies have shown the association between radiomic features and tumor environment [31, 32]. In this study, we performed RNA-seq for a few patients. However, owing to the lack of available data on gene expression or MRI sequences, we were unable to further analyze and validate the association between radiomic features with lncRNAs. In particular, the mechanisms underlying the use of radiomic features to predict recurrence and lncRNA expression need to be further explored. It may be beneficial to combine the RDeepNet model with genetic signatures such as genomics and transcriptomics, which have better prediction for recurrence and clinical application values.

## Conclusions

In conclusion, this study developed and validated a prognostic network that incorporates MRI intratumoral and peritumoral radiomic features for individualized prediction of recurrence risk, which provides an effective tool for survival prediction and clinical decision-making in perioperative patients with nonmetastatic invasive breast cancer. The RDeepNet model was generalized by validation in different breast cancer molecular subtypes and patients treated with endocrine therapy or HER2-targeted therapy. The radiomic features were found to vary among patients with different therapeutic responses and after neoadjuvant chemotherapy. Moreover, the results indicate that radiomics is associated with lncRNAs, and lncRNAs can be quantified by radiomics noninvasively.

### Supplementary Information

**Additional file 1**. Data Supplement Content.

## Data Availability

The novel RNA-seq data used in our study have been deposited in the GEO database, with the accession code of “udilsmwoffixhkr” at https://www.ncbi.nlm.nih.gov/geo/query/acc.cgi?acc=GSE189371. Owing to the privacy of patients, we have only provided the code, tabulated feature value, and expected output as supplementary material approved by the Ethics Committee. Other data related to patients are not available for public access, but MRI datasets and all the code used for analyses following feature extraction from this manuscript are with the Ethics Committee of Sun Yat-sen Memorial Hospital and available from the corresponding author upon reasonable request approved by the Ethics Committee. All experiments and implementation details are described thoroughly in the Methods section and Additional file 1: Data Supplement Content.

## Abbreviations

AUC: Area under the curve
CIs: Confidence intervals
EGFR: Epidermal growth factor receptor
ER: Estrogen receptor
FFPE: Formalin-fixed paraffin-embedded
GLCM: Gray-level co-occurrence matrix
GLDM: Gray-level dependence matrix
GLRLM: Gray-level run-length matrix
GLSZM: Gray-level size zone matrix
GO: Gene Ontology
GSVA: Gene set variation analysis
HER2: Human epidermal growth factor receptor 2
HR: Hormone receptor
HRs: Hazard ratios
KEGG: Kyoto Encyclopedia of Genes and Genomes
LncRNAs: Long non-coding RNAs
MLP: Multilayer neural network
MRI: Magnetic resonance imaging
PR: Progesterone receptor
pTNM: Pathological tumor-node-metastasis
RDeepNet: Radiomic DeepSurv Net
RECIST: Response Evaluation Criteria in Solis Tumors
RFS: Recurrence-free survival
ROIs: Regions of interest
ROC: Receiver operating characteristic curve
T1 + C: Contrast-enhanced T1-weighted imaging
T2WI: T2-weighted imaging
TCGA: The Cancer Genome Atlas
TCIA: The Cancer Imaging Archive
TNBC: Triple-negative breast cancer
NGTDM: Neighboring gray tone difference matrix
