# Supplementary material for: Machine learning radiomics of magnetic resonance imaging predicts recurrence-free survival after surgery and correlation of LncRNAs in patients with breast cancer: a multicenter cohort study
Source: Breast Cancer Res. 2023 Nov 1;25:132. doi: 10.1186/s13058-023-01688-3 (PMC10619251; doi:10.1186/s13058-023-01688-3)
Supplement: Supplementary file 1 — Additional file 1. Data Supplement Content. [file 13058_2023_1688_MOESM1_ESM.docx]

**Data Supplement Content**

**Fig. S1 Performance of the RDeepNet in recurrence risk prediction in patients with different molecular subtypes of cancer.**

**Fig. S2 Overall distribution of radiomic features according to risk stratification from the RDeepNet.**

**Fig. S3** **Association of radiomics with mRNAs.**

**Fig. S4** **Association of radiomics with immune cells.**

**Fig. S5 Overlap of radiomic features according to the association with therapy responses.**

**Fig. S6 Overlap of lncRNAs according to the association with radiomics and RFS.**

**Fig. S7 Performance of the key lncRNAs for predicting the recurrence risk in patients with RNA-seq data.**

**Fig. S8 Performance of the key lncRNAs for predicting the recurrence risk in patients with RNA-seq data.**

**Table S1 Magnetic resonance imaging scanning parameters for the patients.**

**Table S2. Clinicopathological characteristics of patients with RNA-seq data.**

**Table S3 Information of four institutions and clinicopathological characteristics of patients.**

**Table S4 Clinicopathological characteristics of patients in the training, validation, and testing cohorts.**

**Table S5 Classification of therapy-related radiomic features.**

**Table S6 Detailed information of the 15 radiomics- and recurrence-free survival-associated lncRNAs.**

**eAppendix 1 Magnetic resonance imaging acquisition.**

**eAppendix 2 Radiomic feature extraction.**

**Fig. S1 Performance of the RDeepNet in recurrence risk prediction in patients with different molecular subtypes of cancer.**

Kaplan-Meier curves of RFS according to the RDeepNet in the subgroups of (a) luminal A, (b) luminal B, (c) HER2-positive, and (d) triple negative patients. *P* values were calculated using the unadjusted log-rank test; hazard ratios were calculated by univariate Cox regression analysis. CI, confidence interval; HER2, human epidermal growth factor receptor 2; HR hazard ratio; RFS, recurrence-free survival.

**Fig. S2 Overall distribution of radiomic features according to risk stratification from the RDeepNet.**

**
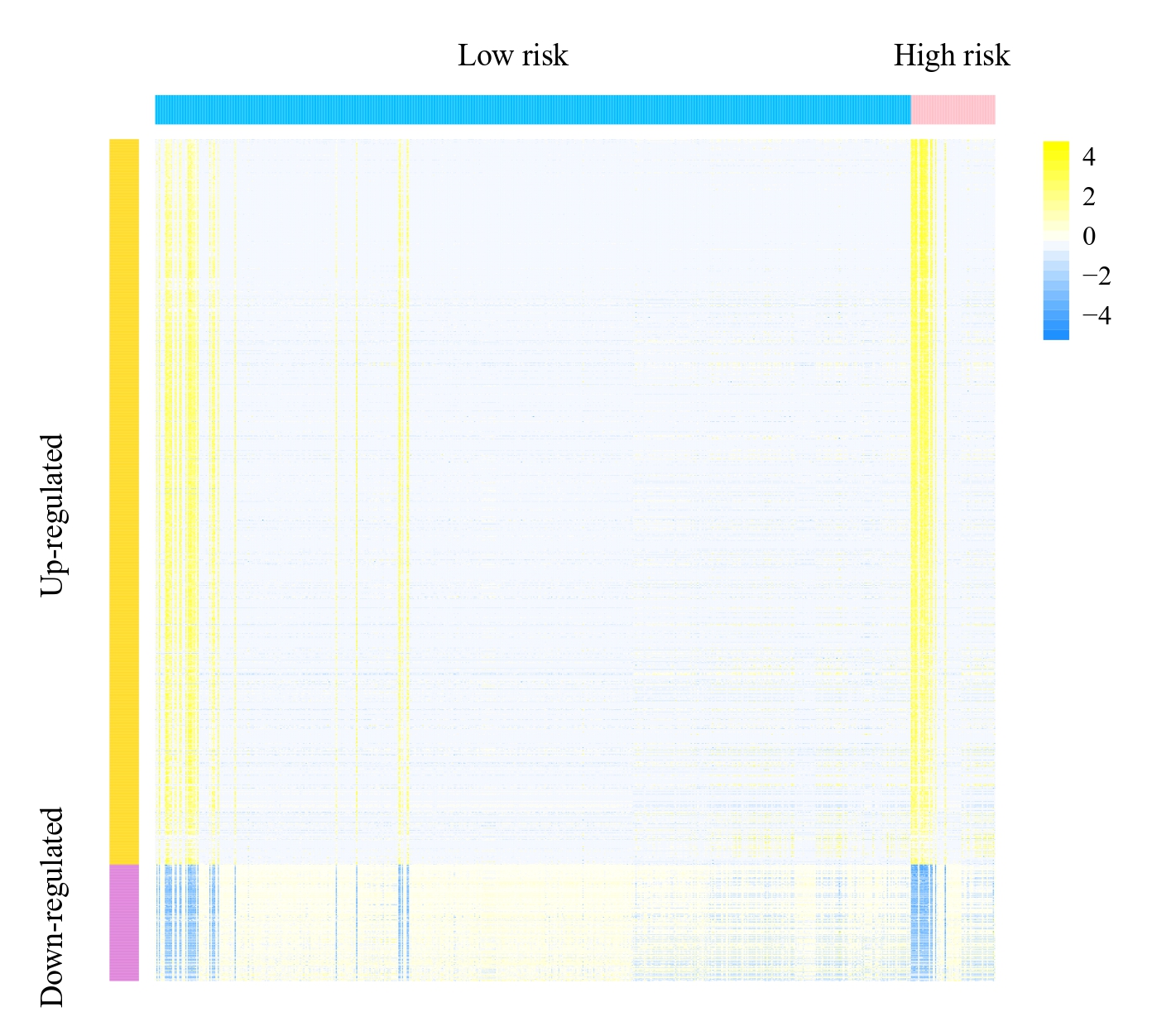
**

Overall distribution of the radiomic features from T1+C, and T2WI sequences in high- and low-risk of recurrence patients from the training cohort. T1+C, contrast-enhanced T1-weighted imaging; T2WI, T2-weighted imaging.

**Fig. S3** **Association of radiomics with mRNAs.**

(a) Differential expression of genes in patients with high or low risk according to the RDeepNet. The KEGG (b), GO (c), and GSVA (d) pathway enrichment analyses of RDeepNet-based genes*.* GO, Gene Ontology; GSVA, gene set variation analysis; KEGG, Kyoto Encyclopedia of Genes and Genomes.

**Fig. S4** **Association of radiomics with immune cells.**

(a) Overall distribution of immune cells in patients with high or low risk according to the RDeepNet. (b) The correlation between RDeepNet score and immune cells. (c) Differential expression of immune cells in patients with high or low risk according to the RDeepNet, **P* < 0.05.

**Fig. S5 Overlap of radiomic features according to the association with therapy responses.**

**
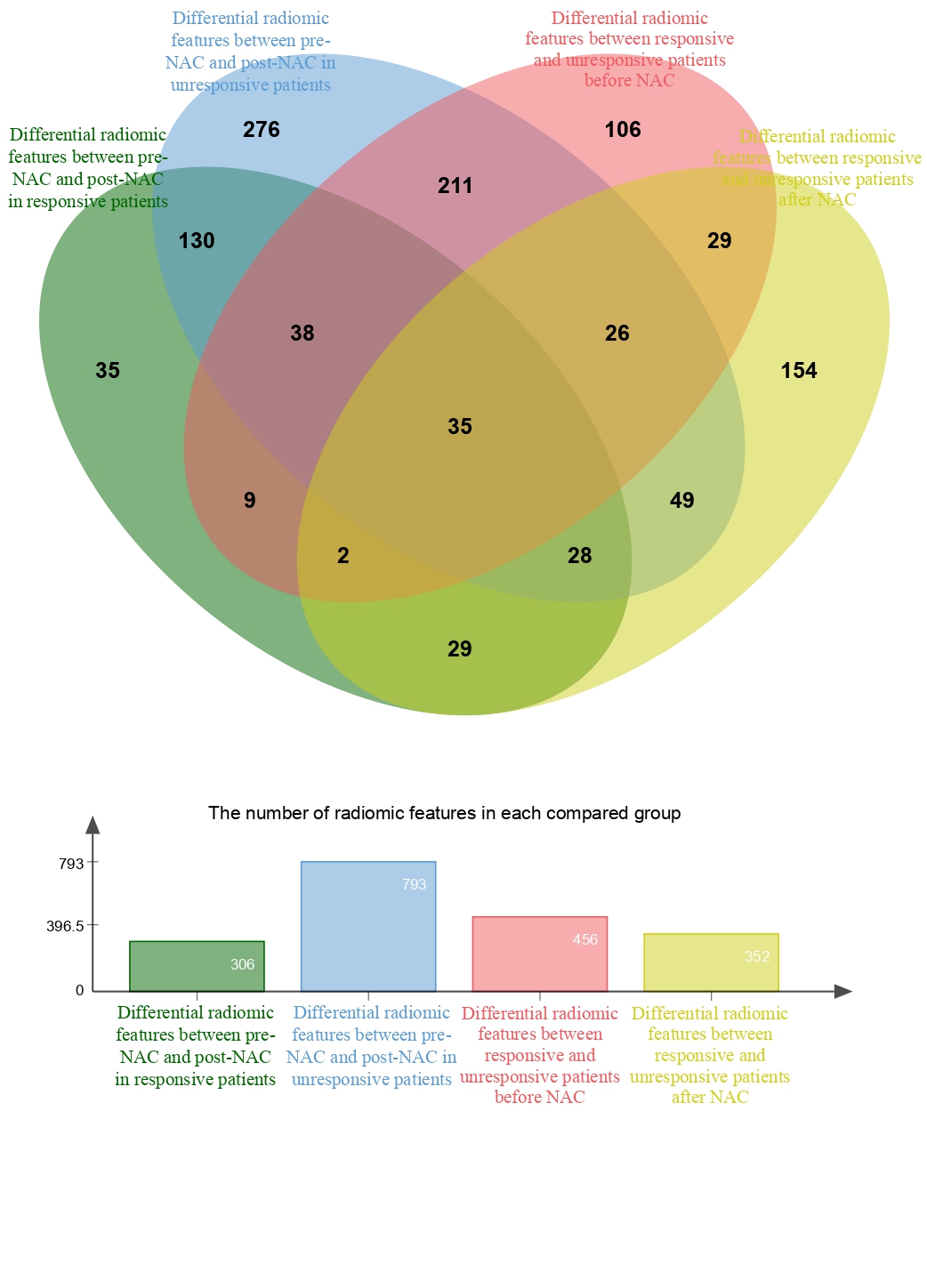
**

*P* values were calculated using the unadjusted log-rank test and paired samples t-test. NAC, neoadjuvant chemotherapy.

**Fig. S6 Overlap of lncRNAs according to the association with radiomics and RFS.**

**
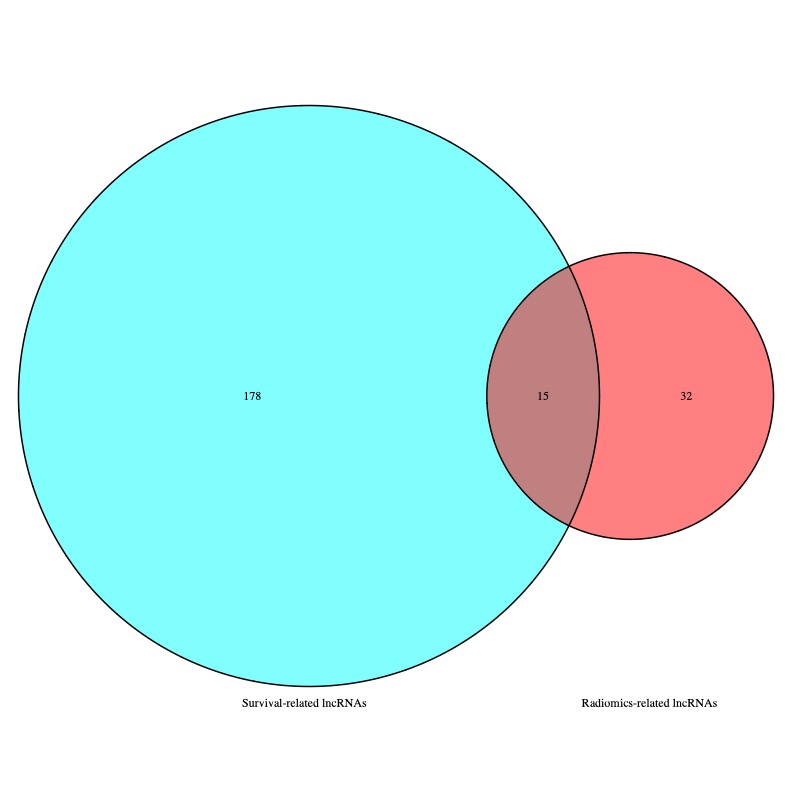
**

*P* values of radiomics-related lncRNAs were calculated using the Spearman rank correlation analysis, *P* values of survival-related lncRNAs were calculated using the unadjusted log-rank test and hazard ratios were calculated by a univariate Cox regression analysis. LncRNAs, long non-coding RNAs; RFS, recurrence-free survival.

**Fig. S7 Performance of the key lncRNAs for predicting the recurrence risk in patients with RNA-seq data.**

Kaplan-Meier curves of RFS according to the expression of lncRNAs (a) AC012467.2, (b) AC093297.2, (c) AC138150.1, (d) AP000253.1, (e) CASC2, (f) DLGAP1-AS2, and (g) LINC00910. *P* values were calculated using the unadjusted log-rank test and hazard ratios were calculated by a univariate Cox regression analysis. CI, confidence interval; HR, hazard ratio; LncRNAs, long non-coding RNAs; RFS, recurrence-free survival.

**Fig. S8 Performance of the key lncRNAs for predicting the recurrence risk in patients with RNA-seq data.**

Kaplan-Meier curves of RFS according to the expression of lncRNAs (a) AC011944.1, (b) AC022034.1, (c) AC025154.2, (d) AC073130.2, (e) AC073367.1, (f) AL122008.4, and (g) NEAT1. *P* values were calculated using the unadjusted log-rank test and hazard ratios were calculated by a univariate Cox regression analysis. CI, confidence interval; HR, hazard ratio; LncRNAs, long non-coding RNAs; RFS, recurrence-free survival.

**Table S1 Magnetic resonance imaging scanning parameters for the patients.**

| Hospital | Scanner | Sequence | TR/TE  (ms) | FOV  (mm) | Matrix | Slice Thickness (mm) | Slice Gap  (mm) | Slices | Flip Angle | Acquisition  Time (min) | Scans |
| --- | --- | --- | --- | --- | --- | --- | --- | --- | --- | --- | --- |
| SYSMH | Philips 1.5T  (Achieva) | T2WI | 4000/60 | 337×240 | 400×318 | 3 | 0 | 55 | 90° | 4min |  |
|  |  | T1+C | 3.3/1.54 | 320×250 | 217×172 | 1 | 0 | 55 | 10° | 7min | 55 |
|  | Philips 3.0T  (Ingenia) | T2WI | 4000/60 | 337×240 | 400×318 | 3 | 0 | 55 | 90° | 4min |  |
|  |  | T1+C | 3.3/1.54 | 320×250 | 217×172 | 1 | 0 | 55 | 10° | 7min | 55 |
|  | Siemens 1.5T  (Avanto) | T2WI | 2760/107 | 350×350 | 320×224 | 5 | 1 | 30 | 150° | 2min46s |  |
|  |  | T1+C | 4.95/2.2 | 380×269 | 288×216 | 3 | 0.6 | 48-72 | 10° | 5-7min | 50/70 |
|  | Siemens 3.0T  (Skyra) | T2WI | 7600/75 | 340×340 | 448×358 | 4 | 0.8 | 35 | 116° | 3min42s |  |
|  |  | T1+C | 3.25/1.22 | 380×327 | 256×218 | 2.5 | 0.5 | 48-72 | 10° | 5-7min | 50/70 |
| SYSUCC | United Imaging 3.0T  (China) | T2WI | 3600/74.34 | 340×340 | 336×335 | 5 | 1 | 24 | 90° | 3min05s |  |
|  |  | T1+C | 4.3/1.99 | 340×340 | 336×335 | 0.67 | 0 | 204 | 10° | 9min58s | 8 |
|  | GE 3.0T  (USA) | T2WI | 3912/107.64 | 380×380 | 416×256 | 5 | 1 | 28 | 111° | 1min59s |  |
|  |  | T1+C | 4.3/1.7 | 360×360 | 256×320 | 1 | 0 | 204 | 5° | 9min50s | 8 |
| SYSUTH | Philips 1.5T  (Achieva) | T2WI | 3400/90 | 260×320 | 348×299 | 3 | 0.3 | 44 | 120° | 4min4s |  |
|  |  | T1+C | 5.4/2.4 | 300×320 | 300×320 | 1 | 0 | 300 | 15° | 7min2s | 6 |
|  | Philips 3.0T  (Ingenia) | T2WI | 4495/70 | 280×340 | 332×377 | 3 | 0 | 52 | 90° | 3min53s |  |
|  |  | T1+C | 4.8/2.1 | 280×340 | 280×339 | 1 | 0 | 300 | 12° | 6min59s | 6 |
| SMUSH | Philips 1.5T  (Achieva) | T2WI | 4518/70 | 320×260 | 256×159 | 4 | 0.07 | 36 | 120° | 3min19s |  |
|  |  | T1+C | 6.0/2.9 | 350×255 | 252×243 | 2 | 1 | 150 | 10° | 6min42s | 9 |
|  | Siemens 3.0T  (Skyra) | T2WI | 4290/78 | 128×332 | 332×128 | 4 | 1 | 30 | 180° | 1min22s |  |
|  |  | T1+C | 5.59/1.96 | 154×338 | 562×256 | 2 | 0.4 | 72 | 10° | 7min15s | 6 |

Abbreviations: FOV, field of view; TR, repetition time; TE, echo time; T1+C, contrast-enhanced T1-weighted imaging; T2WI, T2-weighted imaging.

**Table S2. Clinicopathological characteristics of patients with RNA-seq data.**

|  | Patients with RNA-seq |
| --- | --- |
| Information and characteristics | (No. of patients [n] = 92) |
| Follow-up time, months (median [IQR]) | 36.53 [33.98, 44.50] |
| Age, years (median [IQR]) | 50 [45, 58] |
| Number of tumor (%) |  |
| 1 | 74 (80.4) |
| >1 | 18 (19.6) |
| Tumor size, cm (median [IQR]) | 2.4 [1.8, 3.5] |
| Clinical T stage (%) |  |
| T1 | 30 (32.6) |
| T2 | 53 (57.6) |
| T3 | 7 (7.6) |
| T4 | 2 (2.2) |
| Clinical N stage (%) |  |
| N0 | 48 (52.2) |
| N1 | 43 (46.7) |
| N2 | 1 (1.1) |
| N3 | 0 (0.0) |
| Clinical TNM stage (%) |  |
| I | 21 (22.8) |
| II | 63 (68.5) |
| III | 8 (8.7) |
| Histological grade (%) |  |
| Grade 1 (low) | 5 (5.4) |
| Grade 2 (intermediate) | 38 (41.3) |
| Grade 3 (high) | 49 (53.3) |
| Pathological T stage (%) |  |
| T1 | 51 (55.4) |
| T2 | 38 (41.3) |
| T3 | 3 (3.3) |
| T4 | 0 (0.0) |
| Pathological N stage (%) |  |
| N0 | 45 (48.9) |
| N1 | 23 (25.0) |
| N2 | 13 (14.1) |
| N3 | 11 (12.0) |
| Pathological TNM stage (%) |  |
| I | 31 (33.7) |
| II | 36 (39.1) |
| III | 25 (27.2) |
| ER status (%) |  |
| Negative | 21 (22.8) |
| Positive | 71 (77.2) |
| PR status (%) |  |
| Negative | 38 (41.3) |
| Positive | 54 (58.7) |
| Her2 status (%) |  |
| Negative | 55 (59.8) |
| Positive | 37 (40.2) |
| Ki67 expression (%) |  |
| < 30 | 43 (46.7) |
| ≥ 30 | 49 (53.3) |
| Molecular subtypes (%) |  |
| Luminal A | 11 (12.0) |
| Luminal B | 60 (65.2) |
| Her2-positive | 18 (19.6) |
| Triple negative | 3 (3.3) |
| Type of surgery (%) |  |
| Breast-conserving surgery | 44 (47.8) |
| Others | 48 (52.2) |

Abbreviations: CI, confidence interval; ER, estrogen receptor; Her2, human epidermal growth factor receptor 2; IQR, interquartile range; Ki67, proliferation marker protein Ki-67; PR, progesterone receptor; TNM, tumor–node–metastasis.

**Table S3. Information of four institutions and clinicopathological characteristics of patients.**

|  | Sun Yat-sen  Memorial Hospital | Sun Yat-sen University  Cancer Center | Tungwah Hospital of  Sun Yat-sen University | Shunde Hospital of Southern Medical University |
| --- | --- | --- | --- | --- |
| Information and characteristics | (No. of patients [n] = 682) | (n = 260) | (n = 79) | (n = 92) |
| Investigator in charge | Herui Yao | Chuanmiao Xie | Jie Ouyang | Qiugen Hu |
| Locations | Guangzhou, China | Guangzhou, China | Dongguan, China | Foshan, China |
| Follow-up time, months (median [IQR]) | 43.93 [35.13, 51.60] | 40.43 [30.15, 60.07] | 35.47 [26.05, 48.43] | 56.68 [33.32, 78.31] |
| Age, years (median [IQR]) | 48 [42, 56] | 47 [41, 54] | 46 [43, 52] | 50 [42, 58] |
| Number of tumor (%) |  |  |  |  |
| 1 | 597 (87.5) | 237 (91.2) | 63 (79.7) | 76 (82.6) |
| >1 | 85 (12.5) | 23 (8.8) | 16 (20.3) | 16 (17.4) |
| Tumor size, cm (median [IQR]) | 2.6 [1.9, 3.7] | 2.2 [1.7, 2.8] | 2.1 [1.6, 2.6] | 2.7 [2.1, 3.5] |
| Clinical T stage (%) |  |  |  |  |
| T1 | 201 (29.5) | 111 (42.7) | 37 (46.8) | 21 (22.8) |
| T2 | 394 (57.8) | 133 (51.2) | 37 (46.8) | 64 (69.6) |
| T3 | 77 (11.3) | 9 (3.5) | 3 (3.8) | 5 (5.4) |
| T4 | 10 (1.4) | 7 (2.7) | 2 (2.5) | 2 (2.2) |
| Clinical N stage (%) |  |  |  |  |
| N0 | 341 (50.1) | 157 (60.4) | 72 (91.1) | 59 (64.1) |
| N1 | 314 (46.1) | 90 (34.6) | 6 (7.6) | 17 (18.5) |
| N2 | 22 (3.2) | 13 (5.0) | 0 (0.0) | 13 (14.1) |
| N3 | 4 (0.6) | 0 (0.0) | 1 (1.3) | 3 (3.3) |
| Clinical TNM stage (%) |  |  |  |  |
| I | 145 (21.3) | 83 (31.9) | 36 (45.6) | 18 (19.6) |
| II | 453 (66.5) | 152 (58.5) | 39 (49.4) | 55 (59.8) |
| III | 83 (12.2) | 25 (9.6) | 4 (5.1) | 19 (20.7) |
| Histological grade (%) |  |  |  |  |
| Grade 1 (low) | 37 (5.8) | 5 (1.9) | 6 (8.7) | 3 (3.7) |
| Grade 2 (intermediate) | 321 (50.9) | 131 (50.6) | 36 (52.2) | 42 (51.9) |
| Grade 3 (high) | 273 (43.3) | 123 (47.5) | 27 (39.1) | 36 (44.4) |
| Pathological T stage (%) |  |  |  |  |
| T1 | 363 (53.7) | 131 (50.4) | 32 (40.5) | 36 (39.1) |
| T2 | 275 (40.7) | 119 (45.8) | 45 (57.0) | 49 (53.3) |
| T3 | 27 (4.0) | 10 (3.8) | 1 (1.3) | 5 (5.4) |
| T4 | 11 (1.6) | 0 (0.0) | 1 (1.3) | 2 (2.2) |
| Pathological N stage (%) |  |  |  |  |
| N0 | 352 (51.6) | 147 (56.5) | 44 (55.7) | 47 (51.1) |
| N1 | 211 (30.9) | 64 (24.6) | 25 (31.6) | 21 (22.8) |
| N2 | 75 (11.0) | 31 (11.9) | 6 (7.6) | 14 (15.2) |
| N3 | 44 (6.5) | 18 (6.9) | 4 (5.1) | 10 (10.9) |
| Pathological TNM stage (%) |  |  |  |  |
| I | 219 (32.3) | 78 (30.0) | 21 (26.6) | 23 (25.0) |
| II | 328 (48.4) | 128 (49.2) | 47 (59.5) | 43 (46.7) |
| III | 131 (19.3) | 54 (20.8) | 11 (13.9) | 26 (28.3) |
| ER status (%) |  |  |  |  |
| Negative | 81 (11.9) | 50 (19.2) | 18 (22.8) | 21 (22.8) |
| Positive | 601 (88.1) | 210 (80.8) | 61 (77.2) | 71 (77.2) |
| PR status (%) |  |  |  |  |
| Negative | 216 (31.7) | 70 (26.9) | 20 (25.3) | 34 (37.0) |
| Positive | 466 (68.3) | 190 (73.1) | 59 (74.7) | 58 (63.0) |
| Her2 status (%) |  |  |  |  |
| Negative | 457 (67.0) | 182 (70.0) | 61 (77.2) | 60 (65.2) |
| Positive | 225 (33.0) | 78 (30.0) | 18 (22.8) | 32 (34.8) |
| Ki67 expression (%) |  |  |  |  |
| < 30 | 392 (57.5) | 124 (47.7) | 36 (45.6) | 64 (69.6) |
| ≥ 30 | 290 (42.5) | 136 (52.3) | 43 (54.4) | 28 (30.4) |
| Molecular subtypes (%) |  |  |  |  |
| Luminal A | 113 (16.6) | 46 (17.7) | 17 (21.5) | 19 (20.7) |
| Luminal B | 490 (71.8) | 168 (64.6) | 47 (59.5) | 53 (57.6) |
| Her2-positive | 47 (6.9) | 20 (7.7) | 6 (7.6) | 11 (12.0) |
| Triple negative | 32 (4.7) | 26 (10.0) | 9 (11.4) | 9 (9.8) |
| Type of surgery (%) |  |  |  |  |
| Breast-conserving surgery | 344 (50.4) | 96 (37.1) | 32 (40.5) | 8 (8.7) |
| Others | 338 (49.6) | 163 (62.7) | 47 (59.5) | 84 (91.3) |
| Endocrine therapy (%) |  |  |  |  |
| Yes | 425 (62.3) | 156 (60.0) | 52 (65.8) | 51 (55.4) |
| No | 257 (37.7) | 104 (40.0) | 27 (34.2) | 41 (44.6) |
| HER2-targeted therapy (%) |  |  |  |  |
| Yes | 225 (33.0) | 78 (30.0) | 18 (22.8) | 32 (34.8) |
| No | 457 (67.0) | 182 (70.0) | 61 (77.2) | 60 (65.2) |

Abbreviations: CI, confidence interval; ER, estrogen receptor; Her2, human epidermal growth factor receptor 2; IQR, interquartile range; Ki67, proliferation marker protein Ki-67; PR, progesterone receptor; TNM, tumor–node–metastasis.

**Table S4. Clinicopathological characteristics of patients in the training, validation, and testing cohorts.**

|  | Training  cohort | Validation  cohort | Testing  cohort |
| --- | --- | --- | --- |
| Characteristics | (No. of patients [n] = 698) | (n = 171) | (n = 244) |
| Follow-up time, months (median [IQR]) | 44.67 [34.03, 57.30] | 40.37 [29.32, 62.30] | 39.85 [36.10, 50.88] |
| Age, years (median [IQR]) | 48 [42, 56] | 48 [43, 56] | 46 [40, 53] |
| Number of tumor (%) |  |  |  |
| 1 | 617 (88.4) | 139 (81.3) | 217 (88.9) |
| >1 | 81 (11.6) | 32 (18.7) | 27 (11.1) |
| Tumor size, cm (median [IQR]) | 2.2 [1.7, 3.0] | 2.4 [1.8, 3.2] | 3.5 [2.6, 5.0] |
| Clinical T stage (%) |  |  |  |
| T1 | 288 (41.3) | 58 (33.9) | 24 (9.8) |
| T2 | 371 (53.2) | 101 (59.1) | 156 (63.9) |
| T3 | 29 (4.2) | 8 (4.7) | 57 (23.4) |
| T4 | 10 (1.4) | 4 (2.3) | 7 (2.9) |
| Clinical N stage (%) |  |  |  |
| N0 | 441 (63.2) | 131 (76.6) | 57 (23.5) |
| N1 | 236 (33.8) | 23 (13.5) | 168 (69.1) |
| N2 | 20 (2.9) | 13 (7.6) | 15 (6.2) |
| N3 | 1 (0.1) | 4 (2.3) | 3 (1.2) |
| Clinical TNM stage (%) |  |  |  |
| I | 217 (31.1) | 54 (31.6) | 11 (4.5) |
| II | 433 (62.0) | 94 (55.0) | 172 (70.8) |
| III | 48 (6.9) | 23 (13.5) | 60 (24.7) |
| Histological grade (%) |  |  |  |
| Grade 1 (low) | 25 (3.6) | 9 (6.0) | 17 (8.7) |
| Grade 2 (intermediate) | 341 (49.0) | 78 (52.0) | 111 (57.2) |
| Grade 3 (high) | 330 (47.4) | 63 (42.0) | 66 (34.0) |
| Pathological T stage (%) |  |  |  |
| T1 | 367 (52.6) | 68 (39.8) | 127 (53.4) |
| T2 | 305 (43.7) | 94 (55.0) | 89 (37.4) |
| T3 | 23 (3.3) | 6 (3.5) | 14 (5.9) |
| T4 | 3 (0.4) | 3 (1.8) | 8 (3.4) |
| Pathological N stage (%) |  |  |  |
| N0 | 413 (59.2) | 91 (53.2) | 86 (35.2) |
| N1 | 189 (27.1) | 46 (26.9) | 86 (35.2) |
| N2 | 55 (7.9) | 20 (11.7) | 51 (20.9) |
| N3 | 41 (5.9) | 14 (8.2) | 21 (8.6) |
| Pathological TNM stage (%) |  |  |  |
| I | 238 (34.1) | 44 (25.7) | 59 (24.6) |
| II | 351 (50.3) | 90 (52.6) | 105 (43.8) |
| III | 109 (15.6) | 37 (21.6) | 76 (31.7) |
| ER status (%) |  |  |  |
| Negative | 97 (13.9) | 39 (22.8) | 34 (13.9) |
| Positive | 601 (86.1) | 132 (77.2) | 210 (86.1) |
| PR status (%) |  |  |  |
| Negative | 190 (27.2) | 54 (31.6) | 96 (39.3) |
| Positive | 508 (72.8) | 117 (68.4) | 148 (60.7) |
| Her2 status (%) |  |  |  |
| Negative | 488 (69.9) | 121 (70.8) | 151 (61.9) |
| Positive | 210 (30.1) | 50 (29.2) | 93 (38.1) |
| Ki67 expression (%) |  |  |  |
| < 30 | 362 (51.9) | 100 (58.5) | 154 (63.1) |
| ≥ 30 | 336 (48.1) | 71 (41.5) | 90 (36.9) |
| Molecular subtypes (%) |  |  |  |
| Luminal A | 118 (16.9) | 36 (21.1) | 41 (16.8) |
| Luminal B | 488 (69.9) | 100 (58.5) | 170 (69.7) |
| Her2-positive | 50 (7.2) | 17 (9.9) | 17 (7.0) |
| Triple negative | 42 (6.0) | 18 (10.5) | 16 (6.6) |
| Type of surgery (%) |  |  |  |
| Breast-conserving surgery | 337 (48.3) | 40 (23.4) | 103 (42.2) |
| Others | 360 (51.6) | 131 (76.6) | 141 (57.8) |
| Endocrine therapy (%) |  |  |  |
| Yes | 446 (63.9) | 103 (60.2) | 135 (55.3) |
| No | 252 (36.1) | 68 (39.8) | 109 (44.7) |
| HER2-targeted therapy (%) |  |  |  |
| Yes | 210 (30.1) | 50 (29.2) | 93 (38.1) |
| No | 488 (69.9) | 121 (70.8) | 151 (61.9) |
| RFS rate, % (95 CI) |  |  |  |
| 1-year | 98.5 (97.7-99.4) | 98.2 (96.2-100.0) | 97.5 (95.6-99.5) |
| 2-year | 95.2 (93.6-96.9) | 97.5 (95.1-100.0) | 95.4 (92.7-98.1) |
| 3-year | 93.6 (91.7-95.5) | 96.7 (93.8-99.6) | 93.3 (90.0-96.6) |

Abbreviations: CI, confidence interval; ER, estrogen receptor; Her2, human epidermal growth factor receptor 2; IQR, interquartile range; Ki67, proliferation marker protein Ki-67; PR, progesterone receptor; RFS, recurrence-free survival; TNM, tumor–node–metastasis.

**Table S5 Classification of therapy-related radiomic features.**

| Radiomic features class | Intratumoral region | | |
| --- | --- | --- | --- |
|  | T1+C | T2WI |  |
| Shape | 0 | 0 |  |
| First-order | 2 | 5 |  |
| GLCM | 11 | 3 |  |
| GLSZM | 0 | 0 |  |
| GLDM | 0 | 2 |  |
| GLRLM | 5 | 6 |  |
| NGTDM | 0 | 1 |  |

Abbreviations: T1+C, contrast-enhanced T1-weighted imaging; T2WI, T2-weighted imaging; GLCM, gray-level co-occurrence matrix; GLSZM, gray-level size zone matrix; GLDM, gray-level dependence matrix; GLRLM, gray-level run length matrix; NGTDM, neighboring gray tone difference matrix.

**Table S6 Detailed information of 15 radiomics- and recurrence-free survival-associated lncRNAs**

| LncRNAs | Ensembl ID | Location |
| --- | --- | --- |
| KRT7−AS | ENSG00000257671 | Chromosome 12: 52,245,048-52,247,448 |
| NEAT1 | ENSG00000245532 | Chromosome 11: 65,422,774-65,445,540 |
| DLGAP1-AS2 | ENSG00000262001 | Chromosome 18: 3,603,000-3,610,103 |
| AL122008.4 | ENSG00000236244 | Chromosome 1: 234,268,583-234,272,500 |
| AP000253.1 | ENSG00000234509 | Chromosome 21: 31,653,593-31,659,500 |
| AC025154.2 | ENSG00000257588 | Chromosome 12: 49,951,512-49,962,924 |
| AC012467.2 | ENSG00000271976 | Chromosome 3: 53,858,994-53,861,576 |
| AC138150.1 | ENSG00000224505 | Chromosome 17: 45,150,400-45,161,538 |
| AC073367.1 | ENSG00000225213 | Chromosome 10: 16,721,352-16,748,377 |
| CASC2 | ENSG00000177640 | Chromosome 10: 118,046,279-118,210,158 |
| LINC00910 | ENSG00000188825 | Chromosome 17: 43,338,741-43,389,226 |
| AC022034.1 | ENSG00000237807 | Chromosome 8: 53,493,523-53,524,336 |
| AC011944.1 | ENSG00000177699 | Chromosome 15: 78,978,889-78,985,926 |
| AC093297.2 | ENSG00000272335 | Chromosome 5: 44,826,076-44,828,592 |
| AC073130.2 | ENSG00000243243 | Chromosome 7: 116,237,929-116,327,896 |

Abbreviations: LncRNAs, long non-coding RNAs; ENSG, Ensembl gene.

**eAppendix 1** **Magnetic resonance imaging acquisition.**

In Sun Yat-sen Memorial Hospital of Sun Yat-sen University, patients underwent MRI scan using 1.5T or 3.0T scanners with 8-channel phased-array breast coils. At the scanning, an axial fat-suppressed T2-weighted imaging (T2WI) sequence images were obtained using two b values (0 and 800 s/mm^2^) were acquired before contrast medium administration. An initial fat-saturated T1WI pre-contrast scan was collected before contrast-enhanced T1-weighted imaging (T1+C) images scanning, and then T1+C images were acquired as 50~70 post contrast scans at intervals of 6~8 seconds following the intravenous injection of gadolinium contrast agent. A gadolinium-based agent (Magnevist; Bayer Healthcare, Berlin, Germany) was injected using an MR imaging compatible power injector at a rate of 3.5 ml/s and at a dose of 0.2 ml/kg of body weight, followed by 20 ml saline flush with high-pressure injector.

In Sun Yat-sen University Cancer Center, patients underwent MRI scan using 1.5T or 3.0T scanners with double-breast coils. At the scanning, an axial fat-suppressed T2WI sequence images were obtained using two b values (0 and 800 s/mm^2^) were acquired before contrast medium administration. An initial fat-saturated T1WI pre-contrast scan was collected before T1+C images scanning, and then T1+C images were acquired as eight post contrast scans at intervals of 60 seconds following the intravenous injection of gadolinium contrast agent. A gadolinium-based agent (Magnevist; Bayer Healthcare, Berlin, Germany) was injected using an MR imaging compatible power injector at a rate of 3 ml/s and at a dose of 0.2 ml/kg of body weight, followed by 20 ml saline flush with high-pressure injector.

In Shunde Hospital of Southern Medical University, patients underwent MRI scan using 1.5T or 3.0T scanners with double-breast coils. At the scanning, an axial fat-suppressed T2WI sequence images were obtained using two b values (0 and 1,000 s/mm^2^) were acquired before contrast medium administration. An initial fat-saturated T1WI pre-contrast scan was collected before T1+C images scanning, and then T1+C images were acquired as six postcontrast scans at intervals of 60 seconds following the intravenous injection of gadolinium contrast agent. A gadolinium-basedagent (Gadovist; Bayer Healthcare, Berlin, Germany) was injected using an MR imaging compatible power injector at a rate of 2 ml/s and at a dose of 0.2 ml/kg of body weight, followed by a 20-mlsaline flush with high-pressure injector.

In Tungwah Hospital of Sun Yat-sen University, patients underwent MRI scan using 1.5T or 3.0T scanners with double-breast coils. At the scanning, an axial fat-suppressed T2WI sequence images were obtained using two b values (0 and 800 s/mm^2^) were acquired before contrast medium administration. An initial fat-saturated T1WI pre-contrast scan was collected before T1+C images scanning, and then T1+C images were acquired as six post contrast scans at intervals of 60 seconds following the intravenous injection of gadolinium contrast agent. A gadolinium-based agent (Magnevist; Bayer Healthcare, Berlin, Germany) was injected using an MR imaging compatible power injector at a rate of 2 ml/s and at a dose of 0.2 ml/kg of body weight, followed by 20 ml saline flush with high-pressure injector.

**eAppendix 2 Radiomic feature extraction.**

For all cohorts, multiparametric MR images from all centers were retrieved from Picture Archiving and Communication System, and radiomic features corresponding to the quantitative data obtained after computational translation of images were extracted from MR T1+C, and T2WI imaging. All of the MRIs were normalized to obtain a standard normal distribution of image intensities using the N4ITK Bias Correction code. 3D regions of interest of the breast intratumoral area (ROI-1), and peritumoral area (ROI-2 including the tumor parenchymal constituting 10-mm extension outward) were semi-automatically segmented by 3D Slicer software method (<https://www.slicer.org/>, version 4.10.2). After the ROI-1, and ROI-2 were reconstructed and segmented, the volumes of interest (VOI-1 and VOI-2) images (DICOM format) were transferred to the SlicerRadiomics code, the texture extraction platform developed based on the python package “PyRadiomics”. A total of 3,452 quantitative radiomic features, including six groups of radiomic features were separately extracted from VOI-1 and VOI-2. These included shape, first-order, the gray-level co-occurrence matrix (GLCM), the gray-level size zone matrix (GLSZM), the gray-level dependence matrix (GLDM), and the neigbouring gray tone difference matrix (NGTDM). The normalization was performed on radiomic features using a z-score transformation. All patients were separately reassessed by three radiation oncologists (N Lu, R Zhang and QG Hu ) blinded to the patients’ clinical outcomes, MRI reassessed under the guidance of two senior radiation oncologists (Z Wu and CM Xie) who major in MRI interpretation more than 15 years.
